# Fluorescent and Magnetic Mesoporous Hybrid Material: A Chemical and Biological Nanosensor for Hg^2+^ Ions

**DOI:** 10.1038/srep21820

**Published:** 2016-02-25

**Authors:** Moorthy Suresh, Chokkalingam Anand, Jessica E. Frith, Dattatray S. Dhawale, Vishnu P. Subramaniam, Ekaterina Strounina, Clastinrusselraj I. Sathish, Kazunari Yamaura, Justin J. Cooper-White, Ajayan Vinu

**Affiliations:** 1 Australian Institute for Bioengineering and Nanotechnology (AIBN), Cnr Cooper and College Rd, The University of Queensland, St. Lucia, Queensland 4072, Australia; 2 School of Chemistry and Molecular Biosciences (SCMB), The University of Queensland, St. Lucia, Queensland 4072, Australia; 3 Centre for Advanced Imaging (CAI), Cnr Cooper and College Rd, The University of Queensland, St. Lucia, Queensland 4072, Australia; 4 Superconducting Properties Unit, National Institute for Materials Science, 1-1 Namiki, Tsukuba, Ibaraki 305-0044, Japan; 5 Future Industries Institute, University of South Australia, Mawson Lakes 5095, SA, Australia

## Abstract

We introduce “sense, track and separate” approach for the removal of Hg^2+^ ion from aqueous media using highly ordered and magnetic mesoporous ferrosilicate nanocages functionalised with rhodamine fluorophore derivative. These functionalised materials offer both fluorescent and magnetic properties in a single system which help not only to selectively sense the Hg^2+^ ions with a high precision but also adsorb and separate a significant amount of Hg^2+^ ion in aqueous media. We demonstrate that the magnetic affinity of these materials, generated from the ultrafine γ-Fe_2_O_3_ nanoparticles present inside the nanochannels of the support, can efficiently be used as a fluorescent tag to sense the Hg^2+^ ions present in NIH3T3 fibroblasts live cells and to track the movement of the cells by external magnetic field monitored using confocal fluorescence microscopy. This simple approach of introducing multiple functions in the magnetic mesoporous materials raise the prospect of creating new advanced functional materials by fusing organic, inorganic and biomolecules to create advanced hybrid nanoporous materials which have a potential use not only for sensing and the separation of toxic metal ions but also for cell tracking in bio-separation and the drug delivery.

Mesoporous materials functionalised with transition metal oxides have recently triggered enormous research activities because the d-electrons of these metal oxides, confined to thin walls between pores of mesoporous materials, can provide remarkable magnetic and electrical properties^1^,^2^,^3^,^4^,^5^,^6^. Among the transition metal oxides, iron oxides have received much attention due to their excellent magnetic and adsorptive properties which make them excellent candidates for various applications such as sensing^7^,^8^, adsorption^9^ and magnetic devices^10^,^11^,^12^,^13^. Of particular note, their quick response to a magnetic field offers control over the separation of the solid materials with an external magnet. Another advantage of mesoporous silica functionalised with magnetic metal oxides is the presence of silanol functional groups on the surface that can pave the way for making the inorganic-organic hybrid materials by making covalent connections to organic fluorescent receptors. These hybrid materials, in addition to their excellent optical and magnetic properties, allow dual features in sensing the toxic metals as well as the separation of the same through magnetic approach. However, such materials have only been fabricated in one-dimensional form so far, which severely limits the efficiency of the system for various applications. The introduction of organic-inorganic hybrid functionalities with fluorescent receptors and magnetic metal oxide on the pore channels of 3D silica system promises access to an even wider range of application possibilities. The availability of more adsorption sites in the 3D system aids in better mass transfer and is expected to offer much higher performance in sensing, adsorption or separation of toxic metal ions than the system with a 1D hexagonal structures^14^. In addition, the non-toxicity and fluorescent properties of these materials also provide significant potential in biological applications including cell imaging, intracellular staining and cell tracking^15^,^16^,^17^,^18^,^19^,^20^.

Mercury pollution is regarded as a major threat to the human community due to its high toxicity. Removal of Hg^2+^ ions from aqueous solutions is an important issue in waste water treatment because Hg^2+^ ions are highly toxic even at very low concentrations. Recently, various chemosensors for Hg^2+^ have been designed that can work only in homogeneous media but have the advantages in terms of simplicity, real-time analysis and sensitivity^21^,^22^,^23^,^24^,^25^,^26^,^27^,^28^. However, the use of such homogeneous phases is not suitable for the separation and removal of Hg^2+^ or in rapid screening applications. Recently, a variety of materials capable of removing mercury using inorganic porous solid supports that are non-covalently functionalised with organic dyes in the porous channels have been studied and proposed for adsorption of heavy metals. But the physisorbed dyes limit their durability in long term application^29^. These issues can be overcome by designing a mercury adsorbent/sensor that is covalently functionalised with the fluorescent receptors.

Here we report “sense, track and separate” approach for the removal of Hg^2+^ ion from aqueous media using multifunctional magnetic metal oxide loaded mesoporous silica with a 3D cage type porous structure, covalently functionalised with fluorescent receptors (Fig. 1). The properties of individual components, such as magnetic, fluorescence and optical properties are integrated into the porous system that can perform multiple tasks including sensing, tracking and separation. The extensive internal structure of mesoporous silica was used to carry the non-releasing fluorescent imaging agents, which are stable against enzymatic digestion. We also demonstrate the excellent performance of this novel hybrid system as a sensor that can meet the requirements of Hg^2+^ detection, such as magnetic separation, fluorescence/optical detection and in live cells.

Superparamagnetic FeKIT-5 materials (KIT-Korean Institute of Technology), which have cubic *Fm3m* close-packed symmetry, with different Fe contents were prepared by using polymeric non-ionic surfactant in a highly acidic medium and are denoted as FeKIT-5-x where x denotes the molar *n*_Si_/*n*_Fe_ ratio. The obtained materials possess highly ordered 3D cage type mesopores and ferrosilicate frameworks with ultra-fine Fe_2_O_3_ particles that are uniformly dispersed inside the mesoporous channels. Highly fluorescent rhodamine **R1** (3′,6′-bis(ethylamino)-2′,7′-dimethyl-2-((thiophen-2-ylmethylene)amino)spiro[isoindoline-1,9′-xanthen]-3-one) receptor was covalently immobilised with the silanol groups terminated on the surface of the FeKIT-5 materials in order to sense Hg^2+^ in aqueous media with a high selectivity and adsorption capacity.

## Results and Discussions

### XRD Measurements

**Figure 2:**
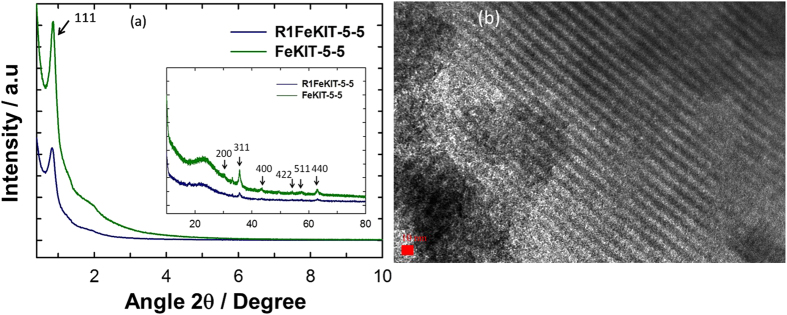
(**a**) Powder X-ray diffraction patterns of FeKIT-5-5 (green) and R1 functionalised FeKIT-5-5 (blue). Inset shows their corresponding higher angle XRD pattern. (**b**) HRTEM image of FeKIT-5-5. 10 nm Scale bar.

The powder XRD patterns of the FeKIT-5 with different Fe ion contents show three diffraction peaks which can be indexed to (111), (200) and (220) reflections of the *Fm3m* symmetry. This confirms the presence of a 3D cubic structure with highly ordered mesopores in the samples and a structure similar to that observed for pure KIT-5 silica (Figs 2, S1 and S2) and the representative XRD patterns of FeKIT-5-5 and R1FeKIT-5-5 are shown in Figs S2 and S3 respectively. Grafting of **R1** into the mesoporous channels of FeKIT-5-5 structure causes a significant reduction in the intensities of the peaks at both low and higher angle peaks which can be ascribed to a large contrast in density between the ferrosilicate walls and the empty pores relative to that between the ferrosilicate walls and the incorporated organic moieties (Fig. 2)^30^,^31^. However, the shape and the width of the peaks were not altered even after the functionalisation, indicating that the structure is quite stable and not affected by the incorporation of the fluorescent molecules inside the nanochannels. The wide-angle XRD patterns of FeKIT-5-5 shows low intense peaks that can be assigned to the typical hkl reflections of approximately at 2θ = 30° (220), 35° (311), 43° (400), 57° (511) and 62° (440) belonged to γ-Fe_2_O_3_ nanoparticles (Supplementary Information Fig. S4)^32^, which is responsible for the magnetic properties. The crystallite size and cage diameter^33^ were calculated to be ca. 17.2 nm and 11.9 nm; respectively. These results, along the TEM images, indicate that the iron oxides particles are formed both in the mesochannels and existence of some γ-Fe_2_O_3_ nanoparticles on the external surface of FeKIT-5-5 material, which has the pore diameter of 5.87 nm. The crystallite size could not be calculated for FeKIT-5-7 due to a low intense 311 reflection peak. However, a low content of iron in the same sample makes it less magnetic than the FeKIT-5-5. The TEM image shown in Fig. 2b confirms the uniform dispersion of iron oxide nanoparticles in most of the places. However, a few large spots (Supporting information Fig. S20) in the image are seen which are attributed to the large iron oxide nanocrystals that are formed on the external surface of the samples.

### N_2_ Adsorption Studies

The low temperature N_2_ adsorption-desorption isotherms of FeKIT-5-5, 7 and 10 materials were of type IV with H2 hysteresis loop, as defined by IUPAC, which is characteristic for the cage type porous materials (Supplementary Information Fig. S1). Table 1 summarises all the structural and microstructural parameters before and after functionalisation of all FeKIT-5 materials. FeKIT5-5, −7, and −10 materials were found to have very high BET surface areas of (907 − 730 m^2^g^−1^), pore volumes (0.40 − 0.37 cm^3^g^−1^) and uniform pore channels (5.87 − 5.94 nm). However, after the functionalisation of these materials with **R1**, the position of the inflection point in the N_2_ isotherm shifted slightly to a low relative pressure values and the volume of N_2_ adsorbed is decreased significantly (Figs S5–S7). This indicates a reduction in pore size and other structural parameters such as surface area (426 − 345 m^2^g^−1^), pore volume (0.19 cm^3^g^−1^) and pore diameter (5.17 − 5.26 nm). The reduction in the structural parameters of the samples after the functionalisation is not due to structural collapse but the presence of **R1** molecules inside the mesochannels of FeKIT-5. It should be noted that the surface area of the support even after the functionalisation is still very high which is critical to adsorb a significant amount of Hg^2+^ ion.

### CP/MAS NMR Measurements

^29^Si magic angle spinning (MAS) and ^1^H−^29^Si cross-polarisation (CP)/MAS nuclear magnetic resonance (NMR) spectroscopy and the Fourier-transform infra-red spectroscopy of the Functionalized samples were performed and the results are shown in Supplementary Information Figs S8–S15. The observed ^29^Si peaks for un-functionalised FeKIT-5 materials were deconvoluted using a least–square fitting using Gaussian normal distribution and the relative population of tetrafunctional species (Q^n^) calculated by the integration of chemical shifts from −91 to −110 ppm that reveal Q^4^, Q^3^, and Q^2^ ratios of 0.64:0.33:0.03, 0.61:0.32:0.07, and 0.6:0.35:0.05 for FeKIT-5-5, FeKIT-5-7, and FeKIT-5-10 respectively. The total concentration of isolated and germinal silanol groups for the same sample was determined to be 6.0, 7.0 and 7.1 mmol/g respectively. For the samples functionalised with (3-chloropropyl)trimethoxysilane (CPTMS), T peaks were clearly observed at −66, −57, −47 ppm in their corresponding ^29^Si MAS spectra which confirmed the formation of tri-functional silicates (−47 ppm (Cl−R−Si (OSi)(OH)_2_), T^1^, −57 ppm (Cl−R−Si (OSi)_2_(OH)), T^2^, and −66 ppm (Cl−R−Si (OSi)_3_), T^3^) and the cross-linking of the CPTMS and the surface silanol groups. The subsequent reaction of FeKIT-5 functionalised with CPTMS to the **R1** also displayed similar kinds of T^3^ and T^2^ peaks but without a T^1^ peak. It can be assumed that absence of T^1^ peaks in **R1** functionalised materials may be due to the condensation of hydroxyl groups in –Si≡(OSi)(OH)_2_ groups with chloro group, assisted by triethylamine used in this reaction through hydrogen bond complex. ^13^C CP/MAS spectrum was also recorded for CPTMS functionalised FeKIT-5 which exhibits chemical shifts at 47, 26 and 10 ppm that are characteristic of Cl−CH_2_−CH_2_−CH_2_−Si(OCH_3_)_3_ and further confirms the introduction of a silyl group as an intact organic moiety. The **R1** functionalised materials also show several sharp peaks at 10.4, 15.2, 26.4, 35.1, 47.1, 58.5, 65.9, 105.7, 128.0, 146.2, 151.3 and 168.0 ppm that confirm the existence of rhodamine fluorophore in its spirolactam form grafted into the mesoporous channels and the chemical stability even after the functionalisation.

### Magnetic Studies

**Figure 3:**
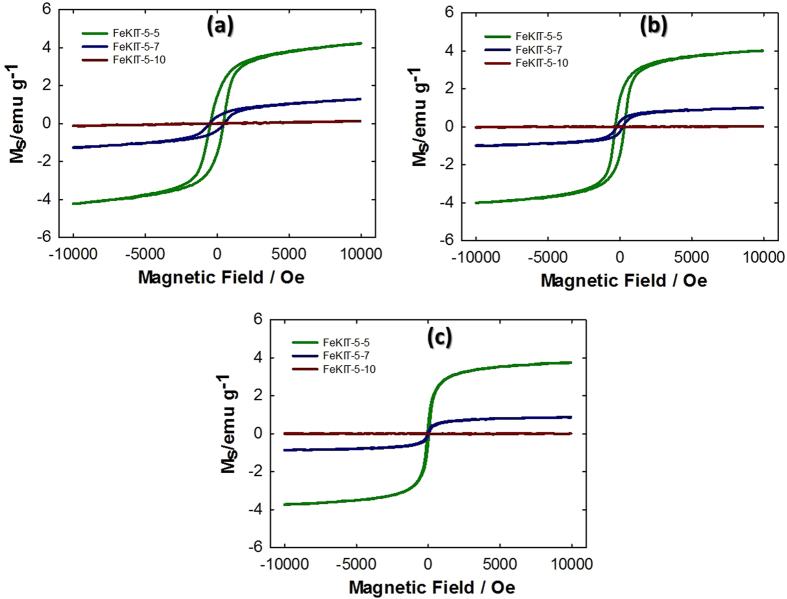
Magnetisation curves for FeKIT-5-5, FeKIT-5-7, and FeKIT-5-10 obtained at (**a**) 10 K, (**b**) 80 K and (**c**) 300 K.

**Figure 4:**
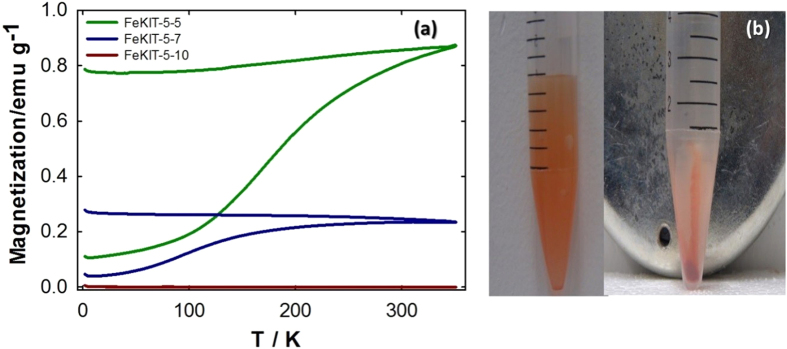
(**a**) Field cooling (FC) and zero field cooling (ZFC) curves of FeKIT-5-5, FeKIT-5-7 and FeKIT-5-10. (**b**) The photographs of the R1FeKIT-5-5 with Hg^2+^ ion in water before and after the external magnetic force.

The magnetic properties of pristine FeKIT-5 materials with different iron contents are shown in Figs 3 and 4. As can be seen in Fig. 3a, the magnetic moment (M_S_) of the samples increases with an increase in the iron contents. In addition, the blocking temperature (T_B_) for the samples varies with the content of the samples. The material with the highest iron content has a largest T_B_ than the materials with the lowest iron content which may be due to the formation of iron oxide particles with different sizes as the iron content is increased. Among the samples studied, FeKIT-5-5 showed the highest saturation magnetic moment (M_S_) and the ferromagnetic behaviour at room temperature. In addition, the Ms of FeKIT-5-5 (4.3 emu/g at 10 K and 3.9 emu/g at 80 K) and FeKIT-5-7 (1.3 emu/g at 10 K and 1.0 emu/g at 80 K) increases with an increase in external magnetic field without reaching saturation up to 1000 Oe. The lack of such magnetic saturation is more significant at 10 K due to the spin canting and various particle size distributions in the mesoporous channels and thus demonstrating the superparamagnetic to ferrimagnetic transition of these materials at low temperature^34^,^35^. However, the hysteresis loop observed at 300 K gave no discernible remanence and coercivity for both FeKIT-5-5 and FeKIT-5-7, indicating a superparamagnetic behaviour from a single domain magnetic material with saturation magnetic moments of 3.7 and 0.9 emu/g, respectively (Fig. 3).

Such superparamagnetic behaviour is known for iron oxide particles smaller than 20 nm^36^ The superparamagnetic behaviour that occurs for such particles in the FeKIT-5-7 materials was supported by the evidences obtained from temperature dependant magnetization investigated under zero field cooling (ZFC). The ZFC curve of FeKIT-5-7 materials decreases slowly with temperature indicating the superparamagnetic behaviour of small particles existed in FeKIT-5-7 material. In contrast to FeKIT-5-7, the ZFC curve obtained for FeKIT-5-5 indicates the constantly increasing trend of magnetisation with temperature up to 300 K suggesting ferromagnetic behaviour at room temperature (Fig. 4).

### Fluorescence Studies

**Figure 5:**
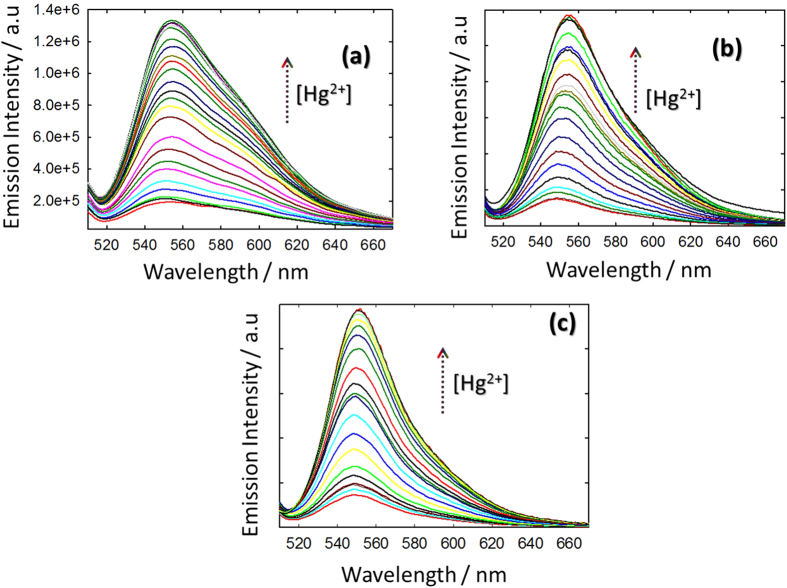
Fluorescence spectra of suspension of 10 mg of (**a**) R1FeKIT-5-5, (**b**) R1FeKIT-5-7 and (**c**) R1FeKIT-5-10 in aqueous solution (pH 7.2) with varying [Hg^2+^] of (**a**) (450 μM) and (**b**) 585 μM and (**c**) 644 μM using λ_ext_ of 500 nm.

Highlighting the utility of these materials for biological applications, an assessment of the specificity of the FeKIT-5 materials for different heavy metals was performed in a biologically-compatible aqueous solution at pH 7.2. The spectra recorded for suspension of 10 mg of FeKIT-5 materials in aqueous phosphate buffer after the addition of various competitive metal ions (50 μM) including Na^+^, K^+^, Ca^2+^, Mg^2+^, Zn^2+,^ Cr^3+^, Cd^2+^, Ni^2+^, Co^2+^, and Hg^2+^ in one minute reveal a very high fluorescence enhancement when only Hg^2+^ was added (Fig. S16). No significant spectral changes occurred in the presence of other metal cations, except Cu^2+^, confirms a high selectivity of FeKIT-5 materials toward the Hg^2+^ due to its thiophilic nature and efficient soft acid-base interactions. The Hg^2+^ ion concentration dependant fluorescence spectra shown in Fig. 5 indicate a gradual increase in fluorescence intensity at 560 nm when excited at 500 nm due to the transformation of non-fluorescent spirolactam form into highly fluorescent xanthane form. The existence of spirolactam form of **R1** in FeKIT-5 materials before addition of Hg^2+^ was supported by a characteristic peak for the C7-atom appeared near 65.9 ppm in their ^13^C CP MAS NMR spectrum. The [Hg^2+^] dependant fluorescence enhancement followed a distinct linearity in the concentration range of 0 to 450 μM, 0 to 585 μM and 0 to 644 μM for **R1** functionalised FeKIT-5-5, FeKIT-5-7 and FeKIT5-10 materials respectively. The increasing order of concentration range is directly related to the incorporation of different amounts of rhodamine incorporated in the FeKIT-5 which follows the order: FeKIT-5-5 <FeKIT-5-7 <FeKIT-5-10. This is mainly due to the difference in the number of silanol group as they dictate the cross-linking of the **R1** molecule. As previously mentioned, among the samples studied, Fe-KIT-5-10 exhibited the highest number of silanol groups together with excellent structural order and textural parameters but with a low magnetic activity. The lower detection limit calculated from the [Hg^2+^] produced fluorescence enhancement three times higher than that of the blank and found to be 0.69, 0.67 and 0.1 ppm respectively.

The Hg^2+^ adsorption isotherm obtained for magnetic R1FeKIT5-5 and R1FeKIT5-7 materials by applying an external strong magnet displayed non-quantitative adsorption at a low concentration and did not give a statistically significant Langmuir adsorption isotherm fit. Significant amounts of residual Hg^2+^ concentration remaining in the solution suggest that the energetically non-uniform existence of rhodamine receptor inside the mesoporous channels provides effective Hg^2+^ ion adsorption. The saturation adsorption capacity of R1FeKIT5-5 and R1FeKIT5-7 was found to be 6.4 mg/g and 9.8 mg/g respectively.

### Confocal Imaging

**Figure 6:**
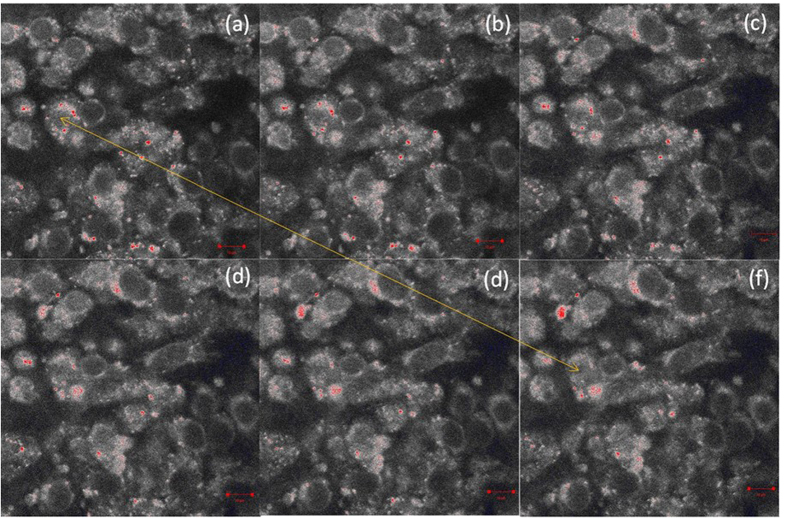
Confocal imaging of 3T3 fibroblasts treated with combinations of R1FeKIT-5-5 (5.0 ppm) and Hg^2+^ (50 μM) showing rhodamine. Time course of 3T3 fibroblasts treated with R1FeKIT-5-5 and Hg^2+^ after application of a magnet from 0 to 10 minutes. The yellow arrow indicates the movement of magnetic particle along the direction of magnet before (**a**) and after (**f**) the application of external magnetic field. Scale bar = 10 μm.

Having proven the effectiveness of the FeKIT-5 materials under physiologically relevant conditions, their utility for the detection of Hg^2+^ in live cells was determined (Fig. 6). Prior to manipulation of nanoparticles in live cells using external magnet, the toxicity of FeKIT-5 materials was tested by MTT assay. FeKIT-5-5 at all concentrations tested caused a decrease in cell viability at 24 hrs as determined by MTT assay. However, after 4 days there was no significant difference between cells treated with FeKIT-5-5 up to 20 ppm and untreated controls. This suggests that some cells are susceptible to FeKIT-5-5 but that the cells which survive the initial treatment are viable and go on to replicate effectively (Supplementary Information Fig. S17). The initial viability of FeKIT-5-5 cells did not differ significantly between 1–20 ppm and so an intermediate concentration of 5 ppm was used for confocal imaging studies to ensure a high signal for imaging and provide numerous particles for tracking. It is however possible that lower concentrations would also be effective and could be used for future studies. The cells that were imaged remained attached to the substrate and were viable for the duration of the imaging experiments.

Confocal imaging of the steady-state fluorescence of NIH3T3 fibroblasts treated with combinations of R1FeKIT-5-5 and Hg^2+^ show bright fluorescence only when labelling of the cells with both R1FeKIT-5-5 and Hg^2+^. No fluorescence was observed in untreated cells or cells treated with either FeKIT-5 or Hg^2+^ separately. This confirms the utility of these hybrid materials for the detection of toxic metal cations adsorbed on cell membrane surfaces in a complex biological system and further demonstrates their potential for future application in the detection and separation of target toxic metal cations. No emission from nucleus of the cell confirms that magnetic particles are not penetrated into the nucleus (Supplementary Information Fig. S18).

As the Ms measured for one of the magnetic materials FeKIT-5-5 showed a high value at room temperature (Ms = 3.7 emug^−1^) compared to other materials. This indicates its ability to be attracted by a small external magnet with a magnetic field of 3000 G. The influence of external applied magnetic field on 3T3 cells, incubated with 50 μM Hg(NO_3_)_2_ for 10 minutes followed by 5.0 ppm R1FeKIT-5-5 for 30 minutes, was studied by confocal imaging to see the movement of magnetic particle inside the cell. It can be seen from the Fig. 6, after applying the external magnet on R1FeKIT-5-5 magnetic particles incubated 3T3 cells show a movement of magnetic particles along on edges of the cells which are located near the magnet.

This figure suggests that manipulation and movement of magnetic particles outside the cell is easier than the particles inside the cell. In order to detect the distance travelled by particle inside the cell, we calculated the euclidean and accumulated distances from 50 different track numbers by using imagej software (Supplementary Information Fig. S19). The maximum and minimum distances were found to be 4.22 μm and 7.95 μm, respectively. Also, its average velocity is 0.1 μm/s. This demonstration of the manipulation of magnetic particles in the cells using an external magnet using FeKIT-5 is a unique example of observing movement of Hg^2+^ ion adsorbed live cells under the influence of external magnetic field using covalently attached fluorescent receptors on mesoporous magnetic materials and gives a significance advance in the emerging field of bionanomaterials and biosensing. With the ability to sense the toxic ions in the living cells or aqueous solution with toxic ions like Hg^2+^, it becomes possible to expand this idea to detect other toxic cations or molecules by modifying the magnetic nanohybrid materials with different structures and fluorophores.

## Conclusions

In conclusion, we have demonstrated the sensing, tracking and separation ability of multifunctional mesoporous hybrid materials, integrated with magnetic and fluorescent properties, towards Hg^2+^ ion in water under physiological conditions. Preliminary confocal imaging experiments showed that these multifunctional mesoporous materials were useful to study the biological processes involving Hg^2+^ in live cells and these new mesoporous materials were successfully utilised for intracellular manipulation of using fluorescent magnetic nanoparticles using a small magnet. Therefore, we believe that these novel multifunctional materials may lead to a new advances and biological application in carrying target specific drugs, bio separation and related processes.

## Experimental Section

### Synthesis of FeKIT-5-X materials

In a typical synthesis, 5.00 g of F127 was dissolved in 240.0 g of distilled water and 3.0 g of HCl (37 wt% HCl). To this mixture, 24.0 g of tetraethyl orthosilicate (TEOS) and the required amount of Fe(NO_3_)_2_.9H_2_O were added under stirring at 45 °C and the mixture was continuously stirred at 45 °C for 24 h. Then, the mixture was kept in an oven at 100 °C for 24 h for hydrothermal treatment under static conditions. The required product was filtered without washing and dried overnight at 100 °C. Finally, the product was calcined at 540 °C to remove the surfactant template. The X in FeKIT-5-X refers the molar ratio of *n*_Si_/*n*_Fe_.

### Synthesis of FeKIT-5-Cl materials

The CPTMS-functionalised FeKIT-5 materials were prepared by using (3-chloropropyl)trimethoxysilane (CPTMS) as silylation reagent. 1.0 g of powder mesoporous silica FeKIT-5, dried under vacuum at 60 °C, was suspended in 50 mL of dry toluene containing 250 μL of CPTMS. The mixture was allowed for stirring for 18 h at 100 °C in a nitrogen atmosphere. The functionalised product was then filtered, washed several times with toluene followed by ethanol, and subjected to Soxhlet extraction in ethanol over the period of 18 h in order to remove residual (ungrafted) organosilane.

### Synthesis of R1FeKIT-5 materials

Hybrid mesoporous material was prepared by reaction of CPTMS-functionalised FeKIT-5 with organic molecule (**R1**). A total of 0.7 g of Fe-KIT-5-Cl was immersed in 25 mL of dry dichloromethane to an adequate disperse, and then (3′,6′-bis(ethylamino)-2′,7′-dimethyl-2-((thiophen-2- ylmethylene)amino)spiro[isoindoline-1,9′-xanthen]-3-one (**R1**) (42.0 mg, 0.08 mmol, 1eq) and dry triethylamine (45.0 μL, 0.32 mmol, 4 eq.) were added and the mixture was stirred for 24 h in nitrogen atmosphere and the resulting product was filtered and washed two times separately with 30 mL of different solvents including dichloromethane, toluene and ethanol.

### Cell Culture

NIH-3T3 mouse fibroblasts (ATCC) were cultured in high glucose DMEM (Gibco/Invitrogen Carlsbad, CA, USA) supplemented with 10% fetal bovine serum ((FBS), Invitrogen) and 1% penicillin/streptomycin (10,000 units Gibco/Invitrogen Carlbad, CA, USA). Cells were maintained at 37 °C in a 5% CO_2_ incubator and passaged upon reaching 90–100% confluence. For imaging, 3T3s were incubated with 50 mM Hg(NO_3_)_2_ for 10 min followed by 10 ppm FeKIT-5 for 30 min. All solutions were made up in culture medium and phosphate buffered saline (PBS) was used to wash the cells after incubation with both Hg(NO_3_)_2_ and R1FeKIT-5. For imaging, the culture medium was replaced with medium without phenol red. Confocal imaging was performed on a Zeiss LSM710 with cell culture chamber (37 °C and 5% of CO_2_).

## Additional Information

**How to cite this article**: Suresh, M. *et al*. Fluorescent and Magnetic Mesoporous Hybrid Material: A Chemical and Biological Nanosensor for Hg^2+^  Ions. *Sci. Rep*. **6**, 21820; doi: 10.1038/srep21820 (2016).

## Supplementary Material

Supplementary Information

## Figures and Tables

**Figure 1:**
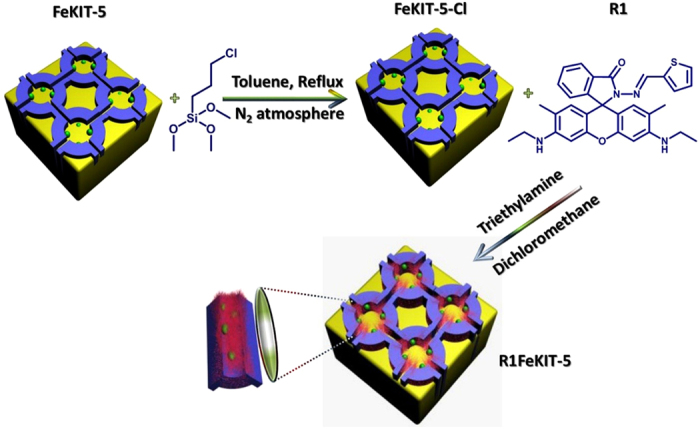
Functionalisation of mesoporous FeKIT-5 materials with (3-chloropropyl)trimethoxysilane (CPTMS) by grafting method and its subsequent covalent functionalisation with R1.

**Table 1:** Textural parameters and magnetic properties of the FeKIT-5 materials synthesised at 100 °C and their corresponding hybrid materials derived from rhodamine derivative (R1).

Sample	A_BET_/(m^2^g^−^)^[a]^	BJH/(nm)^[b]^	Vp/(cm^3^g^−1^)^[c]^	a_0_ (nm)	n_Si_/n_Fe_	Magnetic Measurements^[d]^								
						*M*s [emu g^−1^]			*M*_R_ [emu g^−1^]			*H*_C_ [Oe]		
						10 K	80 K	300 K	10 K	80 K	300 K	10 K	80 K	300 K
FeKIT-5-5	730 ± 3.86	5.87	0.37	18.11	7	4.3	3.9	3.7	1.4	1.4	0.4	529	342	176
FeKIT-5-7	784 ± 5.21	5.92	0.38	18.56	12	1.3	1.0	0.9	0.3	0.2	0.007	511	185	33
FeKIT-5-10	907 ± 5.05	5.94	0.40	18.63	29	–	–	–	–	–	–	–	–	–
R1FeKIT-5-5	345 ± 2.63	5.17	0.19	18.67	7	2.6	2.4	2.2	0.8	0.8	0.3	520	324	122
R1FeKIT-5-7	372 ± 4.28	5.24	0.19	19.95	12	0.9	0.7	0.6	0.3	0.2	0.05	498	172	11
R1FeKIT-5-10	425 ± 3.33	5.26	0.19	20.06	29	–	–	–	–	–	–	–	–	–

[a] Specific surface area calculated by the Brunauer–Emmett–Teller (BJH) method; [b] Pore size derived from adsorption branch by using the Barrett–Joyner–Halenda (BJH) model; [c] Total pore volume estimated from the N_2_ adsorbed amount; [d] Magnetic saturation value (MS), remanence (MR), and coercivity (Hc) obtained from magnetization measurements.
