# Supplementary material for: Fluorescent and Magnetic Mesoporous Hybrid Material: A Chemical and Biological Nanosensor for Hg2+ Ions
Source: Sci Rep. 2016 Feb 25;6:21820. doi: 10.1038/srep21820 (PMC4766400; doi:10.1038/srep21820)
Supplement: Supplementary Information [file srep21820-s1.pdf]

Fluorescent and Magnetic Mesoporous Hybrid Material: A Chemical and Biological Nanosensor for Hg^{2+} Ions

Moorthy Suresh,*[1,2] Chokkalingam Anand,[1] Jessica E. Frith,*[1] Dattatray S. Dhawale,[1] Vishnu P. Subramaniam,[1] Ekaterina Strounina,[3] Clastinrusselraj I. Sathish,[4] Kazunari Yamaura,[4] Justin J. Cooper-White[1] and Ajayan Vinu*[1,5]

[1]Australian Institute for Bioengineering and Nanotechnology (AIBN), Cnr Cooper and College Rd, The University of Queensland, St. Lucia, Queensland 4072, Australia.

[2]School of Chemistry and Molecular Biosciences (SCMB), The University of Queensland, St. Lucia, Queensland 4072, Australia.

[3]Centre for Advanced Imaging (CAI), Cnr Cooper and College Rd, The University of Queensland, St. Lucia, Queensland 4072, Australia.

[4]Superconducting Properties Unit, National Institute for Materials Science, 1-1 Namiki, Tsukuba, Ibaraki 305-0044, Japan.

[5]Future Industries Institute, University of South Australia, Mawson Lakes 5095, SA, Australia

Correspondence and requests for materials should be addressed to A.V. (email: ajayan.vinu@unisa.edu.au)

Table of content

No	Details	Page No
1	Experimental Details	S3-4
2	N_2 adsorption/desorption isotherms FeKIT-5 Materials	S5
3	Low angle powder XRD of FeKIT-5 Materials	S6
4	Low angle powder XRD FeKIT-5 Materials functionalised with R1	S7
5	High angle XRD of FeKIT-5-5	S8
6	Pore size distribution curves of FeKIT-5-5 and R1FeKIT-5-5	S9
7	Pore size distribution curves of FeKIT-5-7 and R1FeKIT-5-7	S10
8	Pore size distribution curves of FeKIT-5-10 and R1FeKIT-5-10	S11
9	^{29}Si CP/MAS NMR spectra for FeKIT-5 Materials	S12
10	^{29}Si CP/MAS NMR spectra for R1FeKIT-5-5 Material	S13
11	^{29}Si CP/MAS NMR spectra of FeKIT-5 materials functionalised with CPTMS	S14
12	^{13}C CP/MAS NMR spectra of FeKIT-5-5 grafted with CPTMS	S15
13	^{13}C CP/MAS NMR spectra of FeKIT-5-7 grafted with CPTMS	S16
14	^{13}C CP/MAS NMR spectra of FeKIT-5-10 grafted with CPTMS	S17
15	^{13}C CP/MAS NMR spectra of R1FeKIT-5-5	S18
16	IR Spectra of FeKIT-5 materials	S19
17	Steady-State fluorescence scanning experiment with different metal cations	S20
18	Confocal images	S21
19	Distance Measurement	S22-23
20	MTT assay	S24
21	TEM images	S25

1. Experimental details.

Materials:

Surfactant Pluronic F127, ethylene oxide (EO)/propylene oxide (PO) triblock copolymer, composition of $EO_{106}PO_{70}EO_{106}$, with the average molecular weight of 12600 and 2-thiphenecarbaldehyde, (3-Chloropropyl)trimethoxysilicane (CPTMS), Rhodamine-6G, tetraethyl orthosilicate (TEOS), $Fe(NO_3)_3$. $9H_2O$ and all metal salts were purchased from Aldrich. All chemicals were of analytical grade and were used without further purification.

Methods:

XRD: Powder X-ray diffraction patterns were collected on a Rigaku diffractometer using CuKα (λ = 0.15406 nm) radiation, operated at 40 kV and 40 mA. The diffractograms were recorded in the 2θ range of 0.7 to 80 ° with a 2θ step size of 0.01 ° and a step time of 10 second.

TEM: The HRTEM images were obtained with TEM JEOL JEM-2100F. The preparation of samples for HRTEM analysis involved sonication in ethanol for 5 min and deposition on a copper grid. The samples were imaged at an accelerating voltage of 200 kV.

Magnetic Measurements: The dc magnetization measurements were performed with a SQUID magnetometer (MPMSXL, Quantum Design). Zero-field cooling (ZFC) and Field-Cooling (FC) measurements were carried out in the following way: First at zero field the sample was cooled down to 7K from room temperature. Measurements were then conducted at various fields (100, 500, and 1000 Oe) as the temperature was increased from 7 to 300K. FC measurements were performed immediately after ZFC measurement from 300 to 7 K under the same applied field. The magnetic hysteresis measurements were conducted at 5, 15, and 25 K between -1 and 1T and also under the influence of smaller Magnetic Fields of ±2.5T.

N_2 Adsorption studies: Nitrogen adsorption–desorption isotherms at 77K were determined in a Micromeritics 2040 sorptometer. The samples (FeKIT-5 and R1FeKIT-5) were outgazed for 12 hours at 250°C and 100°C respectively, under vacuum condition. The surface area measurements were carried out according to the Brunauer–Emmett–Teller (BET) method. The pore size distributions were calculated with the DFT Plus software (Micromeritics), applying the Barrett–Joyner–Halenda (BJH) model with cylindrical geometry of the pores.

Fluorescence and confocal imaging experiments: Room temperature steady state luminescence spectra were recorded with HORIBA JOBIN YVON spectrophotometer. Confocal images of organic-dye-labeled magnetic materials were obtained with a Zeiss LSM 510 CLSM equipped with a computer-controlled scan stage. An argon laser for RITC excitation at 543 nm (488 nm for FITC) was used for imaging.

ICP analysis: was carried out using a Varian (brand) Vista Pro (model) ICPOES instrument operating at 1000 W forward power and measuring mercury at the 184.887 nm analytical line. The sample was introduced to the ICPOES using an all – quartz torch and spray chamber system incorporating a Glass Expansion (brand) conical (model) nebuliser (2 mL/min volume) and a cyclonic spray chamber.

Hg^{2+} adsorption studies: 5 mg portions of the functionalized mesostructure encapsulates (R1FeKIT5-5 and R1FeKIT-5-7) were dispersed in 5 ml of mercury(II) nitrate solutions at initial concentrations ranging from 0 to 70 ppm at 25°C for 24 hours. After magnetic separation, the remaining concentration of Hg^{2+} ion was measured by ICP analysis to obtaine adsorption isotherms by plotting the amount of Hg adsorbed per gram of R1FeKIT5-5 and R1FeKIT-5-7 as a function of total amount of Hg(II) per gram of adsorbent.

The adsorption isotherms were fitted by the Langmuir model using the following equation.

$$Qe = abCe / (1 + bCe)$$

where Q*e* is the equilibrium adsorption capacity (mg g^{-1}), C*e* is the concentration after equilibration, *b* is the Langmuir constant that directly relates to the adsorption affinity (L mg^{-1}), *a* is the saturation capacity (mg g^{-1}).

Synthesis of FeKIT-5 Materials, Rhodamine 6G hydrazone (I), and R1:

Synthesis of FeKIT-5 materials: In a typical synthesis, 5.00 g of F127 was dissolved in 240 g of distilled water and 3 g of HCl (37 wt % HCl). To this mixture, 24.0 g of tetraethyl orthosilicate (TEOS) and the required amount of $Fe(NO_3)_2.9H_2O$ were added under stirring at 45 °C and the mixture was continuously stirred at 45 °C for 24 h. Then, the mixture was kept in an oven at 100 °C for 24 h for hydrothermal treatment under static conditions. The required product was filtered without washing and dried overnight at 100 °C. Finally, the product was calcined at 540 °C to remove the surfactant template.

Rhodamine 6G hydrazone (1) and **R1** were prepared following a literature method.[1]

Rhodamine-6G (0.958 g, 2 mmol) was dissolved in 40 mL ethanol and allowed to dissolve completely. To this, 3.0 mL hydrazine monohydrate (80%) was added dropwise with vigorous stirring at room temperature. Finally, the stirred mixture was heated to reflux for 2 h, during which the dark purple solution disappeared and pink precipitate appeared after cooling overnight. The resulting precipitate was filtered and washed 3 times with 15 mL cold EtOH/water. After drying in vacuum, the reaction afforded Rhodamine-6G hydrozone (1).

Rhodamine 6G hydrozide (1; 7.0 mmol, 3 g) and thiophene-2-carbaldehyde (7.0 mmol, 0.854 g) were stirred in boiling methanol with 3 drops of acetic acid. After 2 h of stirring, white precipitates was obtained. This white precipitate was filtered and washed with methanol: ether (1:1) and dried in vaccum.

1. M. Suresh, S. Mishra, S. K. Mishra, E. Suresh, A. K. Mandal, A. Shrivastav, A. Das *Org. Lett.*, **2009**, *11*, 2740.

2. N_2 adsorption/desorption isotherms FeKIT-5 Materials

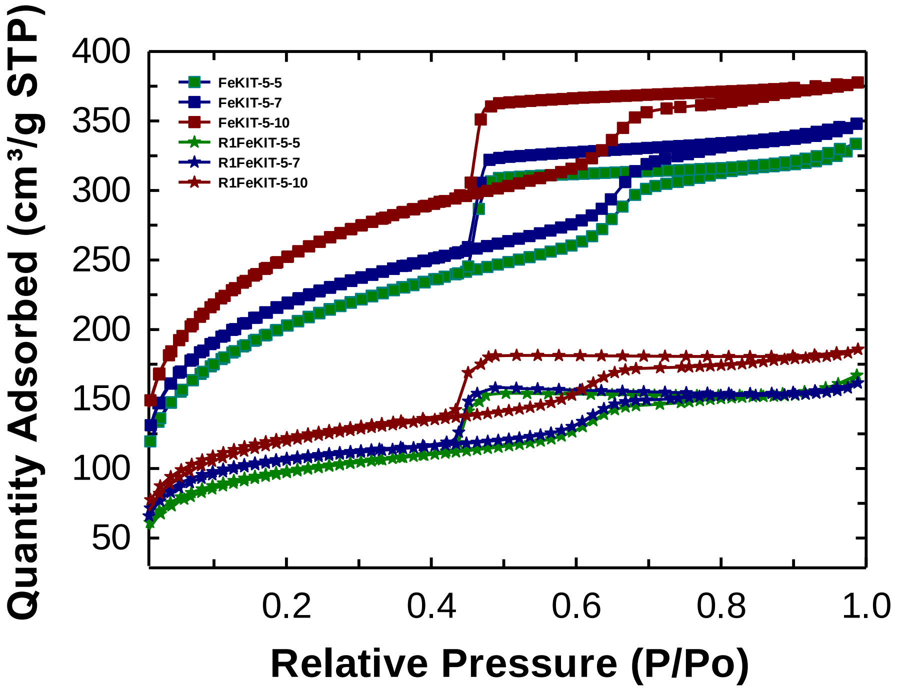

Figure S1. N_2 adsorption/desorption isotherms of FeKIT-5-5, FeKIT-5-7, FeKIT-5-10, R1FeKIT-5-5, R1FeKIT-5-7 and R1FeKIT-5-10.

3. Low angle powder XRD of FeKIT-5 Materials

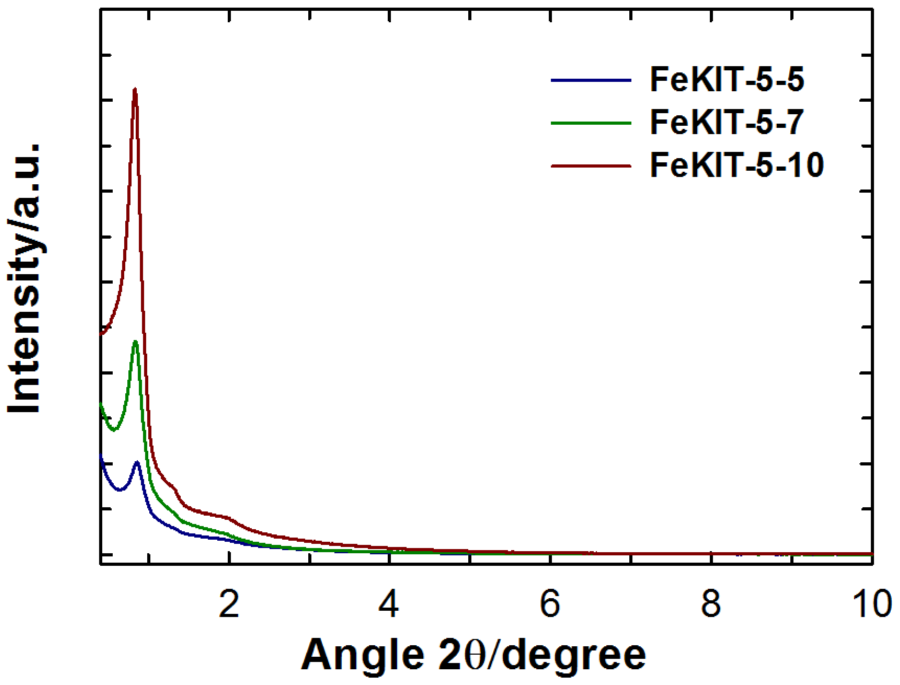

Figure S2. Low angle powder X-ray diffraction patterns of FeKIT-5-5, FeKIT-5-7 and FeKIT-5-10after calcination at 540 °C.

4. Low angle powder XRD FeKIT-5 Materials functionalised with R1

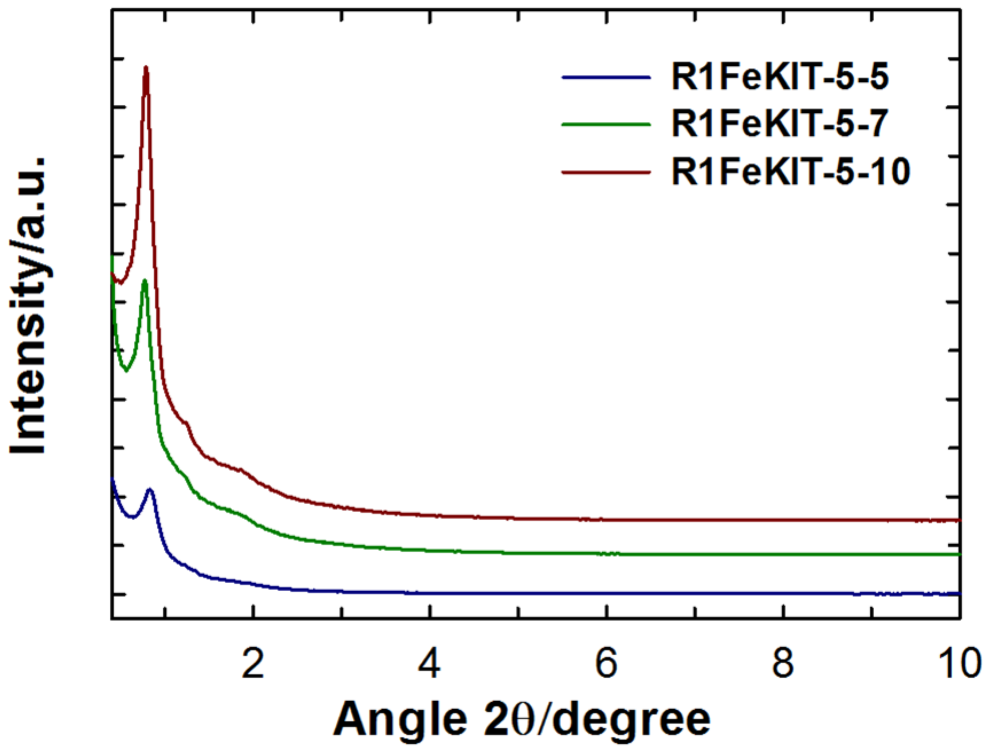

Figure S3. Low angle powder X-ray diffraction patterns of R1FeKIT-5-5, R1FeKIT-5-7 and R1FeKIT-5-10after calcination at 540 °C.

5. High angle XRD of FeKIT-5-5

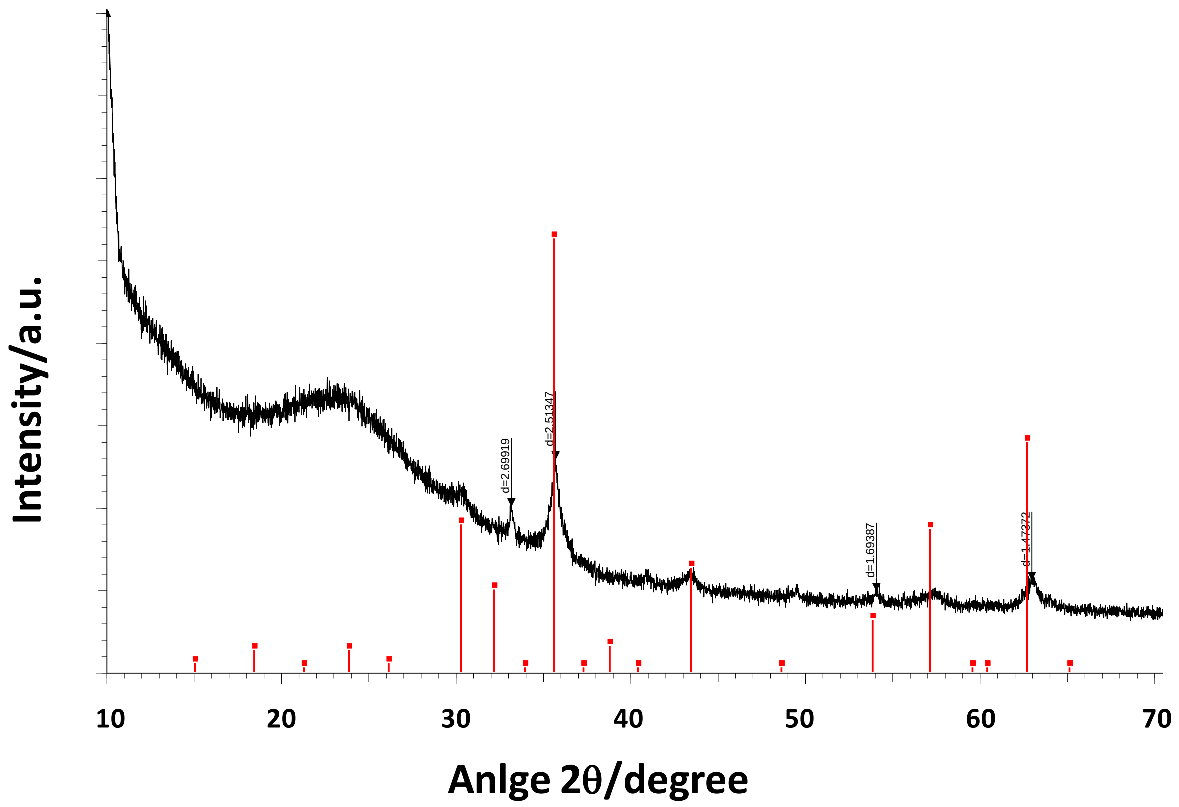

Figure S4. Comparison high angle powder X-ray diffraction patterns of FeKIT-5-5 with γ-Fe_2O_3 phase.

6. Pore size distribution curves of FeKIT-5-5 and R1FeKIT-5-5

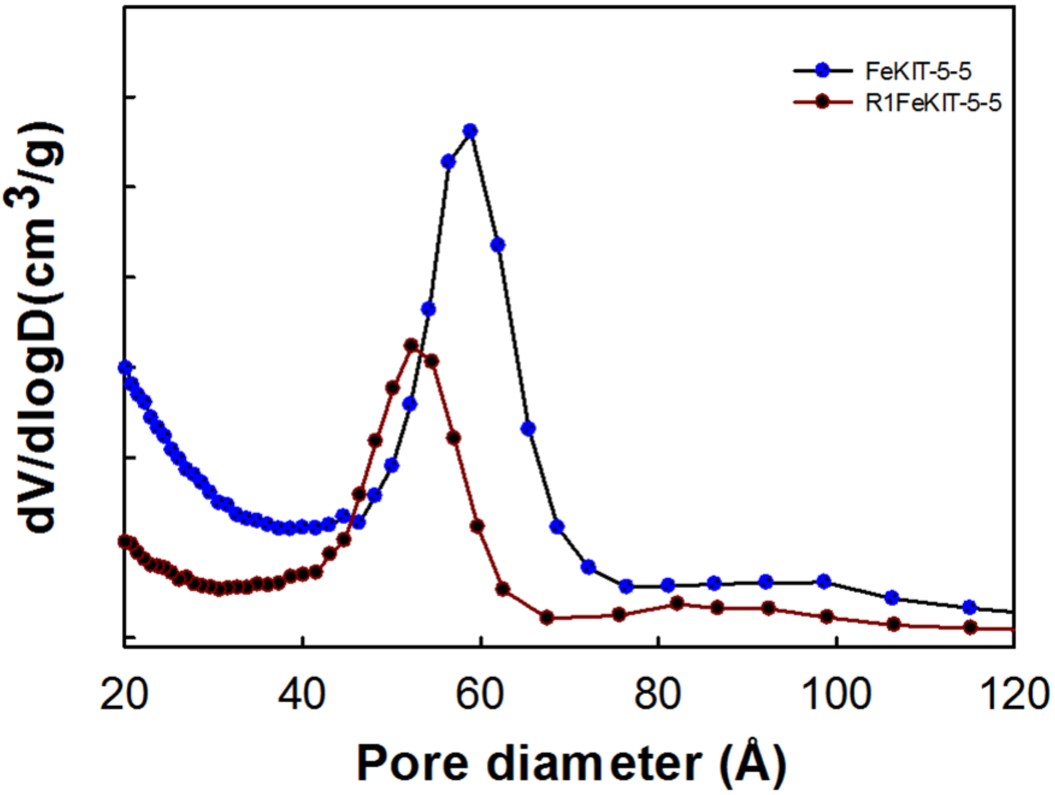

Figure S5. Pore size distribution curves of FeKIT-5-5 and R1FeKIT-5-5.

7. Pore size distribution curves of FeKIT-5-7 and R1FeKIT-5-7

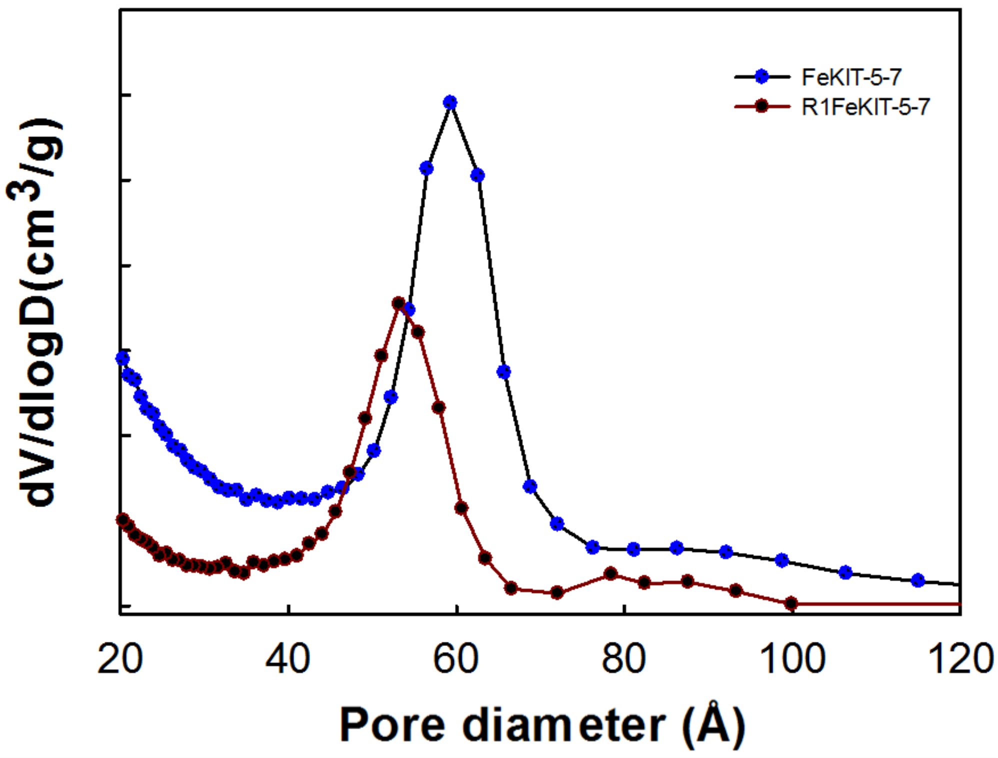

Figure S6. Pore size distribution curves of FeKIT-5-7 and R1FeKIT-5-7.

8. Pore size distribution curves of FeKIT-5-10 and R1FeKIT-5-10

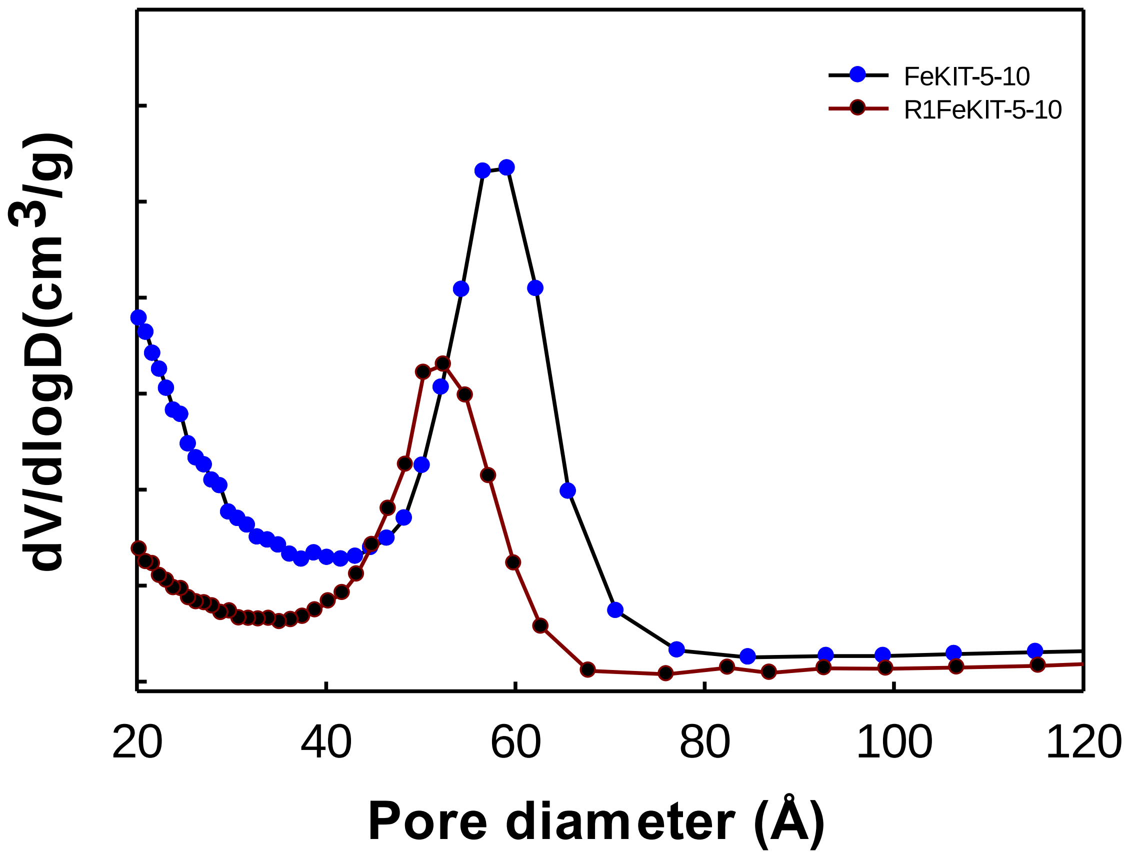

Figure S7. Pore size distribution curves of FeKIT-5-10 and R1FeKIT-5-10.

9. ^{29}Si CP/MAS NMR spectra for FeKIT-5 Materials

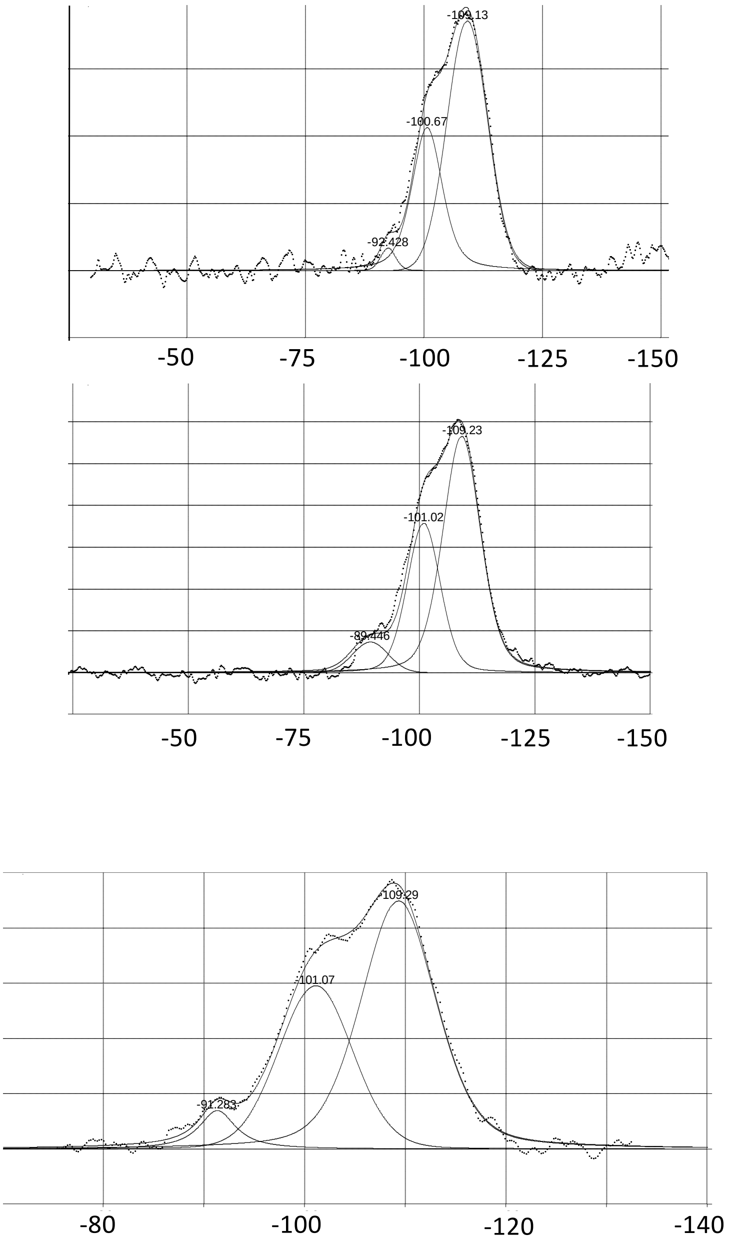

Figure S8. Deconvoluted ^{29}Si CP/MAS NMR spectra of (a) FeKIT-5-5, (b) FeKIT-5-7 and (c) FeKIT-5-10

10. ^{29}Si CP/MAS NMR spectra for R1FeKIT-5-5 Material

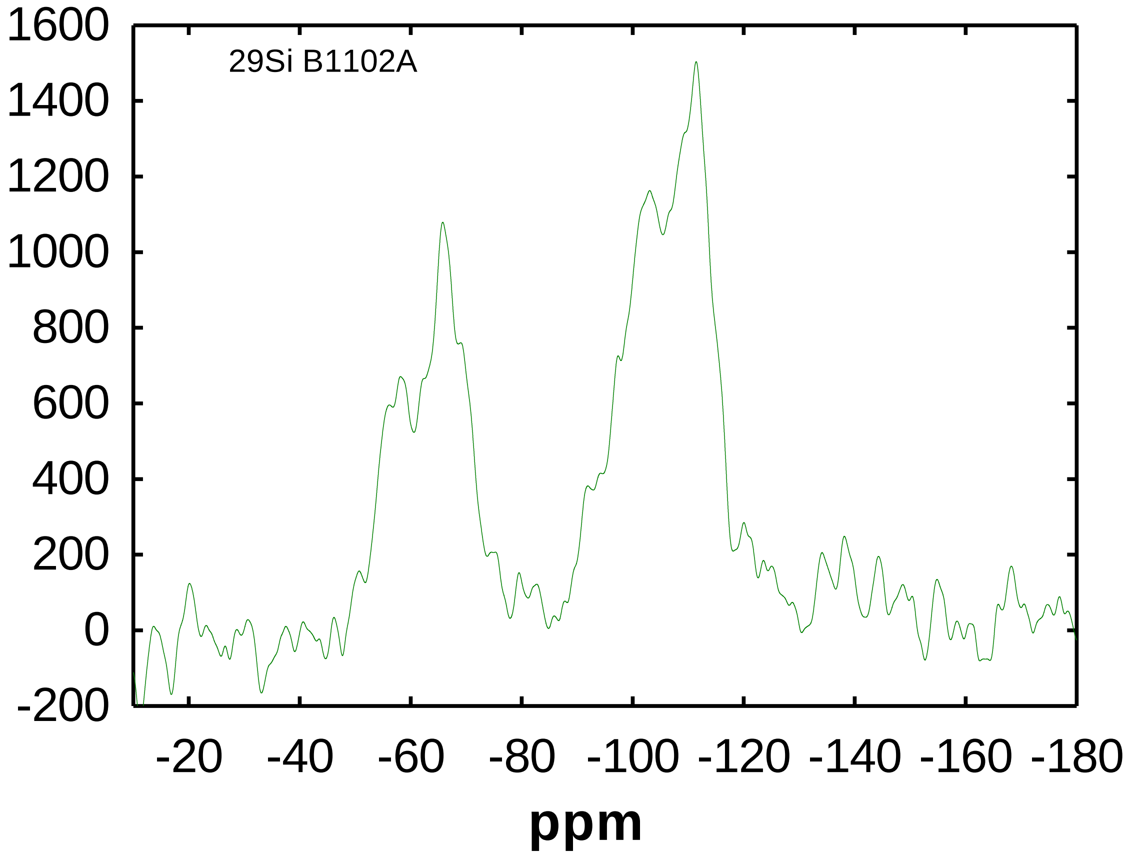

Figure S9. ^{29}Si CP/MAS NMR spectra of R1FeKIT-5-5.

11. ^{29}Si CP/MAS NMR spectra of FeKIT-5 materials functionalised with CPTMS

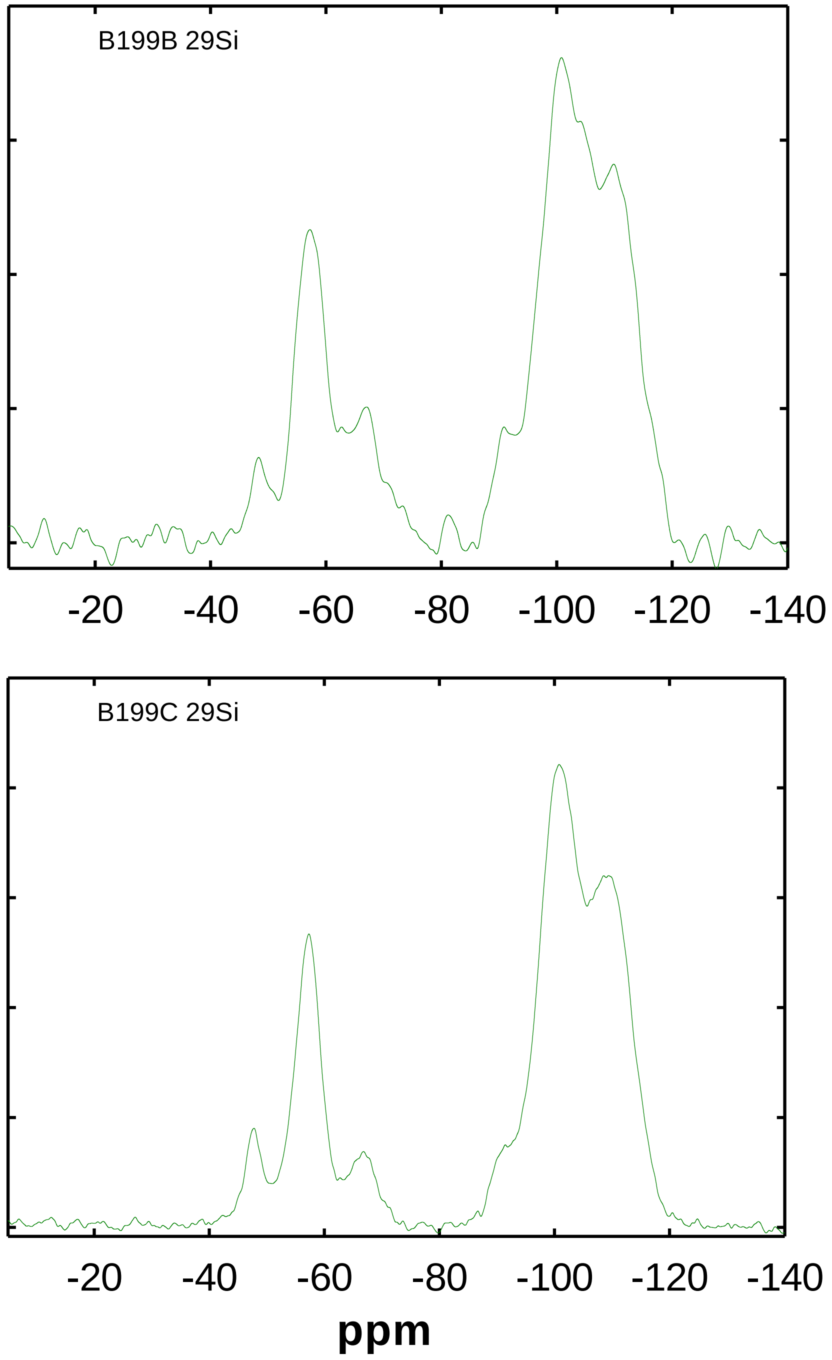

Figure S10. ^{29}Si CP/MAS NMR spectra of FeKIT-5 materials functionalised with 3-chloropropyl trimethoxysilane.

12. ^{13}C CP/MAS NMR spectra of FeKIT-5-5 grafted with CPTMS

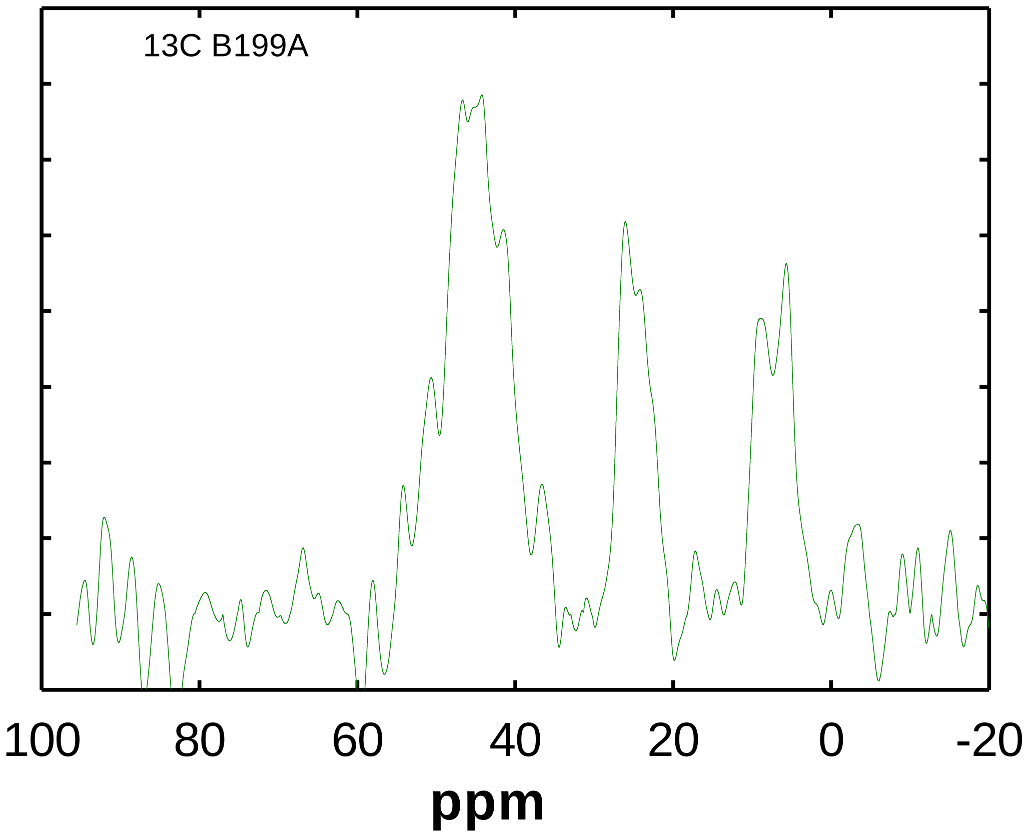

Figure S11. ^{13}C CP/MAS NMR spectra of FeKIT-5-5 grafted with 3-chloropropyl trimethoxysilane.

13. ^{13}**C CP/MAS NMR spectra of FeKIT-5-7 grafted with CPTMS**

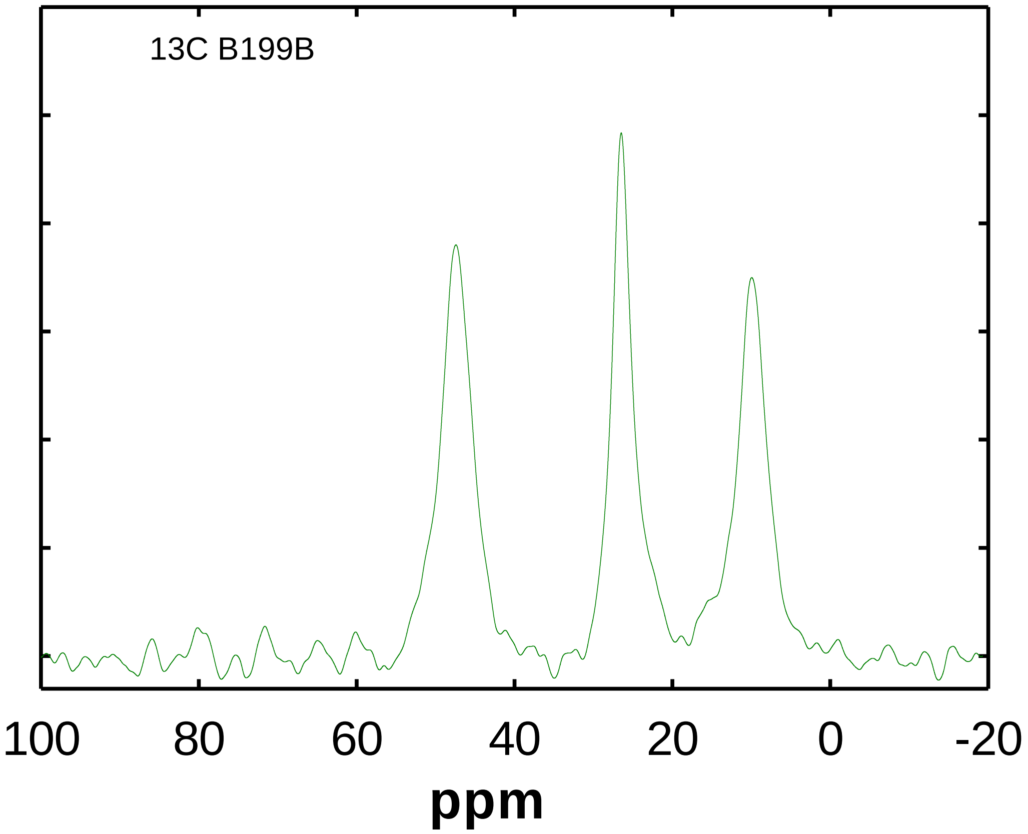

Figure S12. ^{13}C CP/MAS NMR spectra of FeKIT-5-7 grafted with 3-chloropropyl trimethoxysilane.

14. ^{13}C CP/MAS NMR spectra of FeKIT-5-10 grafted with CPTMS

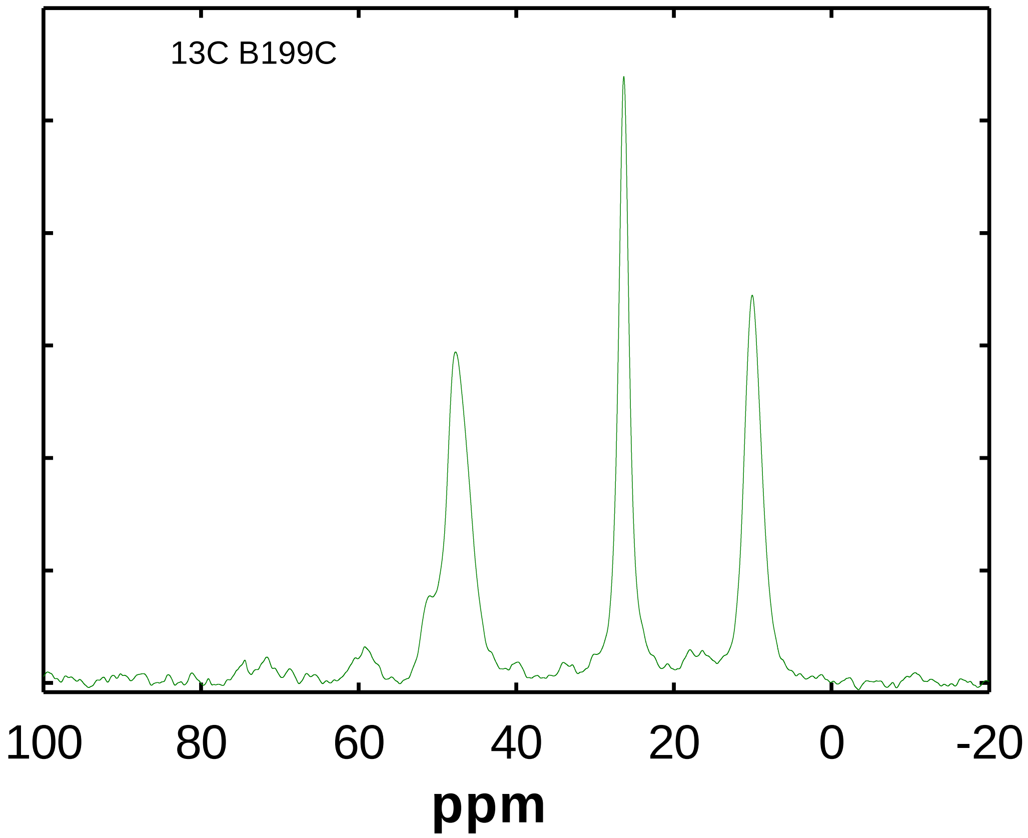

Figure S13. ^{13}C CP/MAS NMR spectra of FeKIT-5-10 grafted with 3-chloropropyl trimethoxysilane.

15. ^{13}C CP/MAS NMR spectra of R1FeKIT-5-5

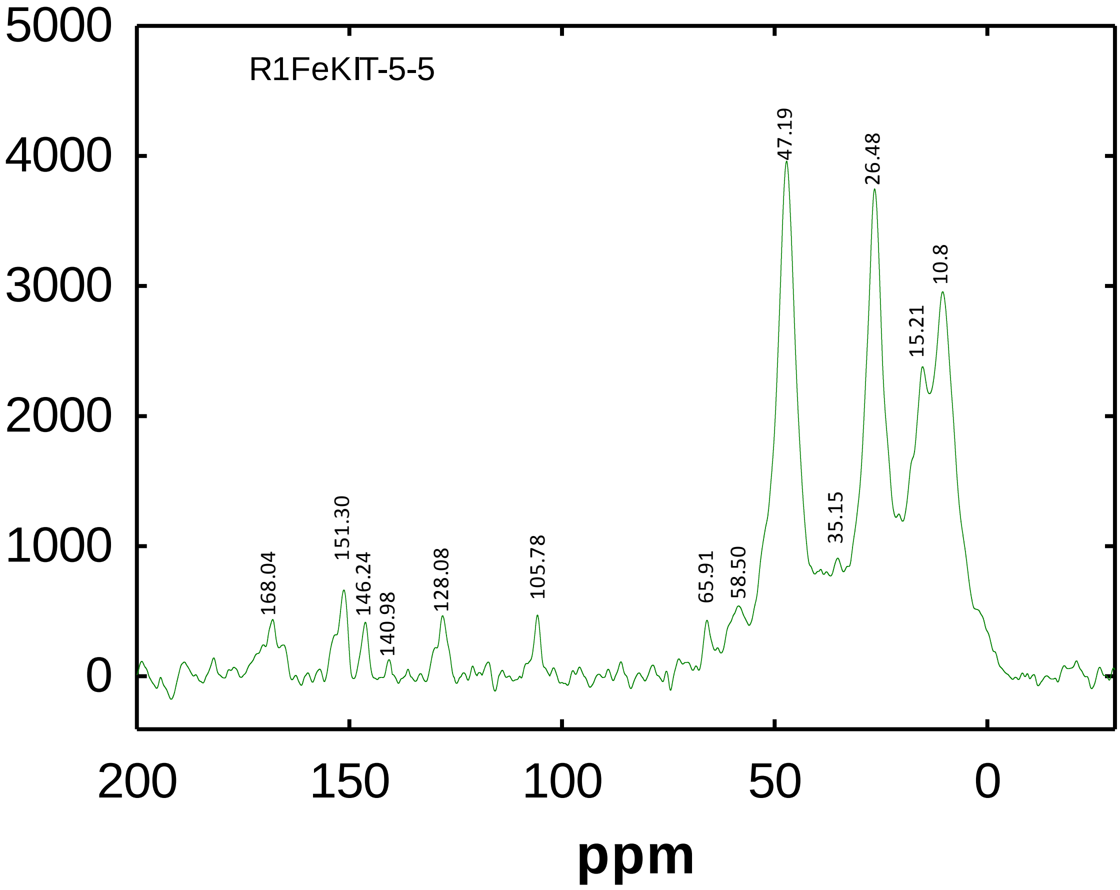

Figure S14. ^{13}C CP/MAS NMR spectra of R1FeKIT-5-10.

16. IR Spectra of FeKIT-5 materials

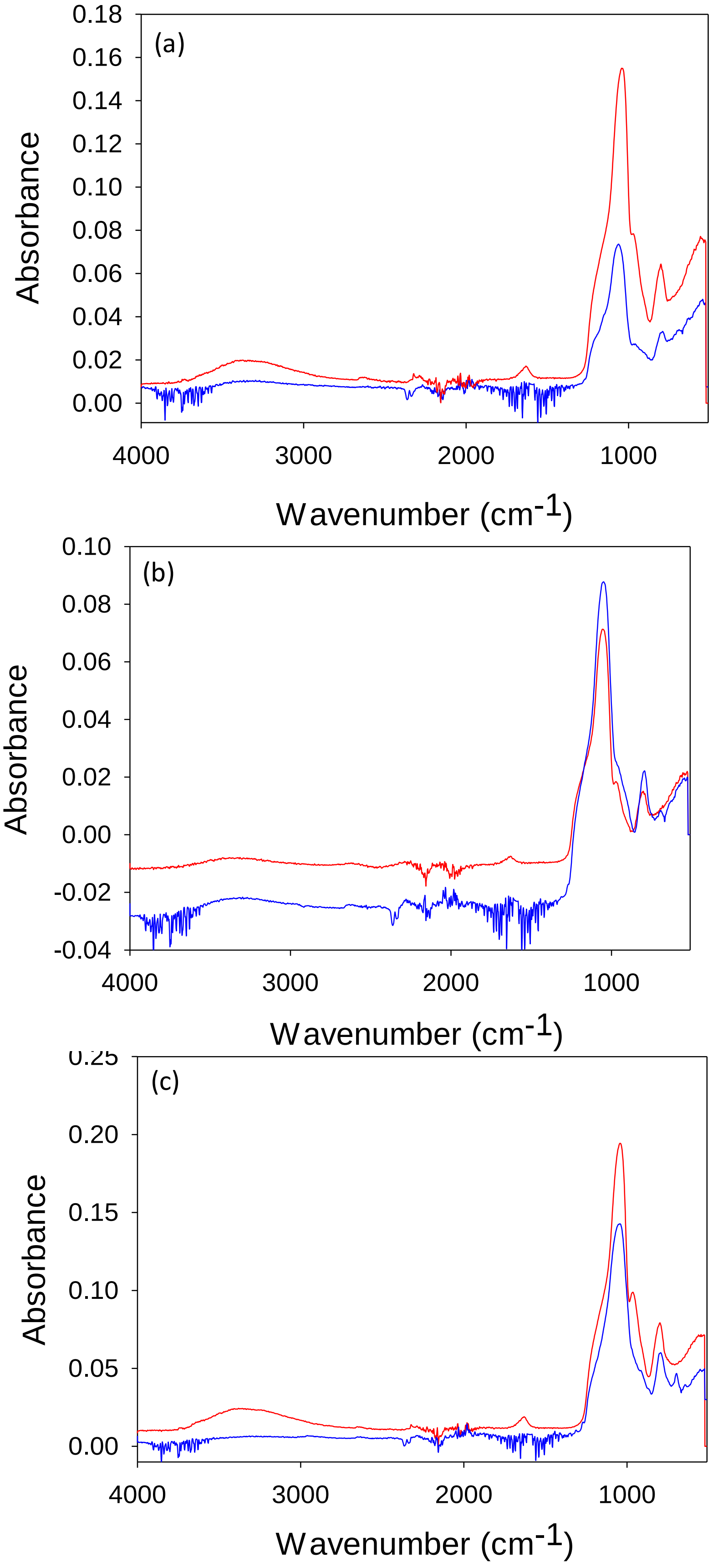

Figure S15. IR Spectra of (a) FeKIT-5-5, (b) FeKIT5-7 and (c) FeKIT5-10 Materials.

17. Steady-State fluorescence scanning experiment with different metal cations:

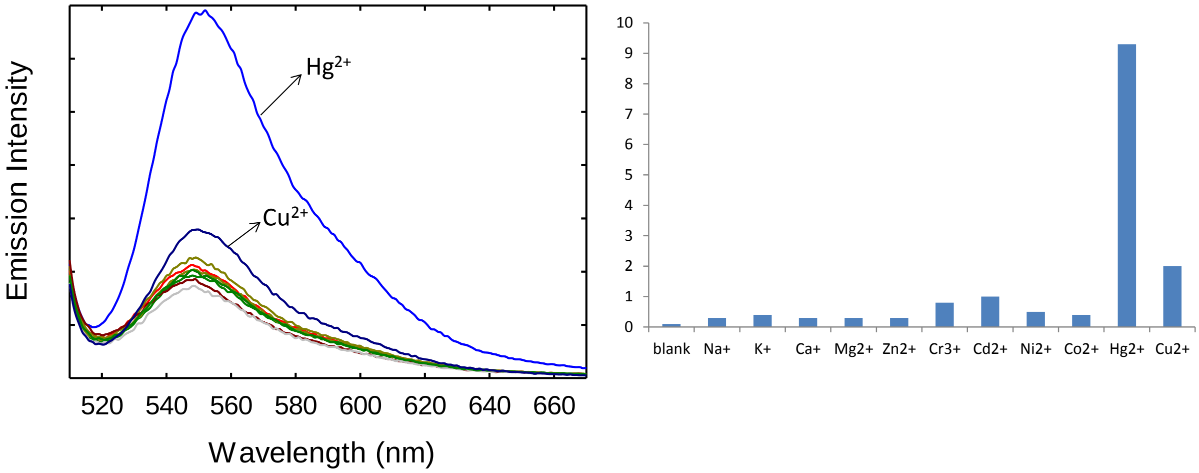

Figure S16. Fluorescence spectra of **R1FeKIT-5-5** (10.0 mg) in water pH 7.2 with respective metal cations (50µM). λ_{ext} at 500 nm.

18. Confocal Images

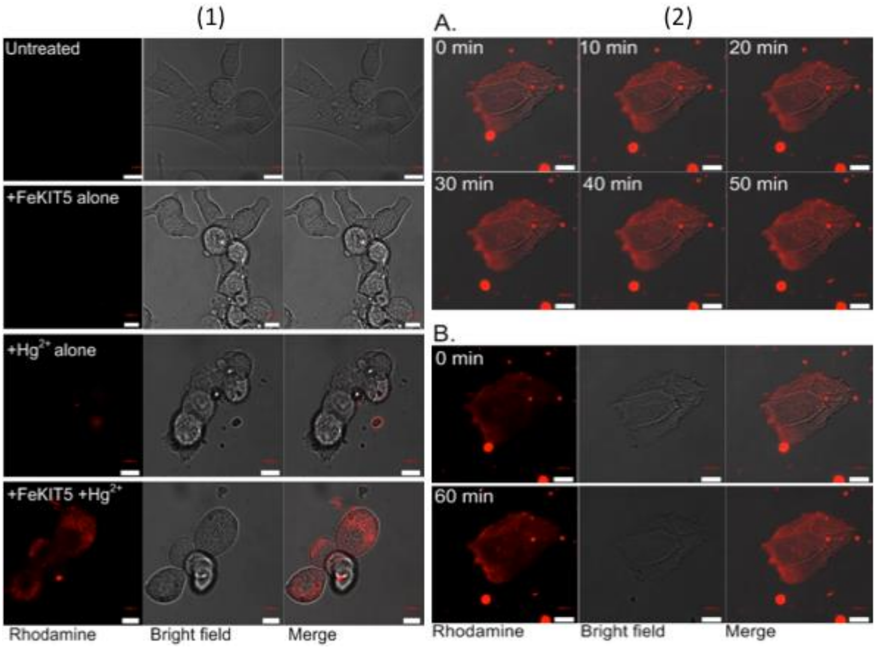

Figure S17. (1) Confocal imaging of 3T3 fibroblasts treated with combinations of R1FeKIT-5-5 and Hg^{2+} showing rhodamine, bright-field images and a merged image. Scale bar = 10μm. (2) Time course of 3T3 fibroblasts treated with R1FeKIT-5-5 and Hg^{2+} after application of a magnet. A) Merge of rhodamine and bright field images at different times after application of a magnet, B) Separate rhodamine and bright-field images of cells at 0 and 60 minutes after application of a magnet. Scale bar = 10μm.

19. Distance measurement

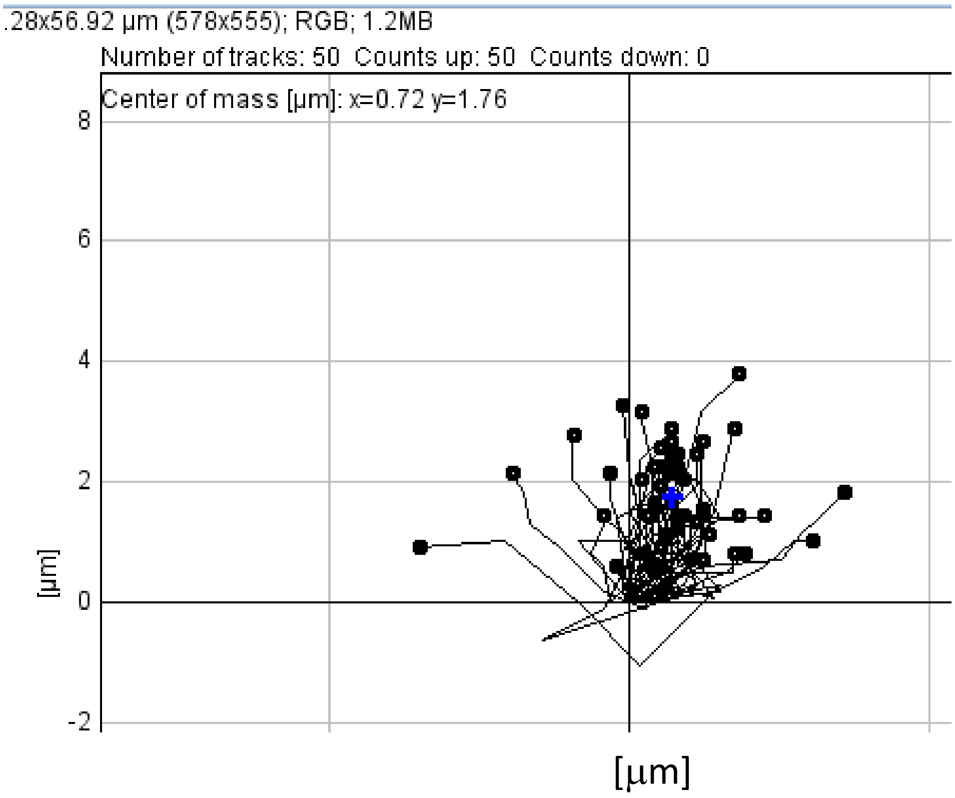

Figure S18. 50 different trajectories used for calculation of average distance traveled by nanoparticles using ImageJ software.

Track number	Velocity (um/sec)	Accumulated distance (um)	Euclidean distance (um)	Directionality
1	0.01	3.18	2.76	0.868
2	0.01	1.88	1.49	0.796
3	0.01	2.16	2.06	0.952
4	0.01	2.39	2.25	0.943
5	0.01	3.41	3.28	0.962
6	0.01	2.69	2.61	0.971
7	0.01	2.18	1.93	0.882
8	0.01	4.03	2.4	0.596
9	0.01	2.04	0.65	0.317
10	0	1.04	0.8	0.773
11	0.03	7.95	3.61	0.454
12	0.01	3.33	3.24	0.973
13	0.01	2.54	0.85	0.332
14	0.01	3.62	1.97	0.544
15	0.01	1.96	1.65	0.844
16	0	1.36	1.29	0.944
17	0.01	2.57	1.06	0.411
18	0.01	3.09	1.69	0.547
19	0.01	3.12	2.56	0.822
20	0.01	2.64	1.59	0.602
21	0.02	4.85	0.85	0.174
22	0.01	1.76	1.75	0.993
23	0.01	1.7	0.66	0.387
24	0.01	4.24	4.04	0.952
25	0.01	4.46	4.22	0.946
26	0.01	3.37	2.94	0.871
27	0.01	3.63	2.59	0.715
28	0.01	3.08	2.71	0.879
29	0	1.45	1.42	0.984
30	0	1.47	1.25	0.854
31	0.01	2.8	1.79	0.639
32	0.01	2.55	2.34	0.916
33	0.01	3.67	3.36	0.914
34	0.01	3.05	2.02	0.662
35	0.01	3.2	2.67	0.835
36	0.01	2.5	1.47	0.588
37	0.01	2.17	2.11	0.974
38	0.01	2.06	1.89	0.916
39	0.01	1.65	1.48	0.894
40	0.01	2.35	1.75	0.744
41	0.01	2.72	2.59	0.953
42	0.01	1.85	1.71	0.921
43	0.01	3.82	2.92	0.764
44	0.01	2.38	2.34	0.984
45	0.01	2.2	2.18	0.987
46	0.01	3.02	2.9	0.961
47	0.01	3.17	2.96	0.933
48	0.01	3.27	2.27	0.695
49	0.01	3.28	3.19	0.971
50	0.01	2.48	2.29	0.926

Summary

Slice length in dataset: equal to 6
Number of current used tracks: 50

x Forward migration index: 0.3
y Forward migration index: 0.65
Directionality: 0.79

Center of mass
x: 0.72 [µm]
y: 1.76 [µm]
Length: 1.9 [µm]

Accumulated distance
Max Distance: 7.95 [µm]
Min Distance: 1.04 [µm]
Mean Distance: 2.83 [µm] SD: 1.11 [µm]

Euclidean distance
Max Distance: 4.22 [µm]
Min Distance: 0.65 [µm]
Mean Distance: 2.17 [µm] SD: 0.85 [µm]

Velocity
Max Velocity: 0.03 [µm/sec]
Min Velocity: 0.0 [µm/sec]
Mean Velocity: 0.01 [µm/sec] SD: 0.0 [µm]

20. MTT assay

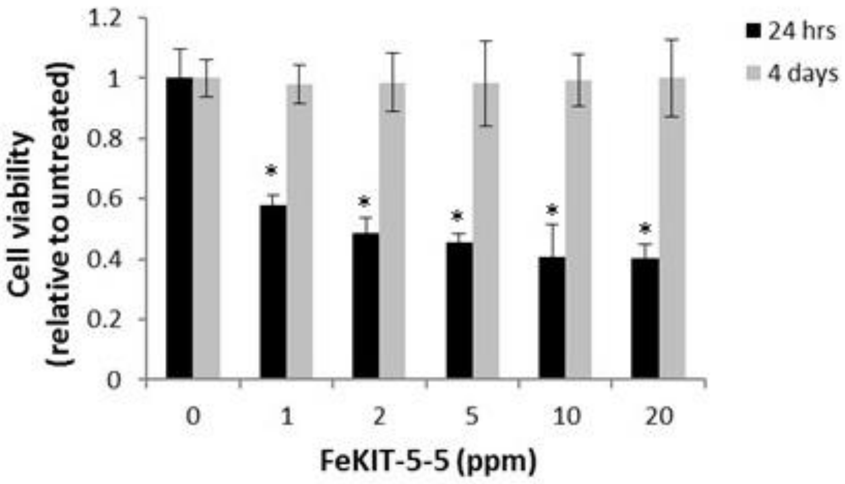

Figure S19. Cell viablity test using MTT assay. Data is shown as mean±SD for N=4 replicates, * denotes $p<0.05$ as determined by t-test.

21. TEM image

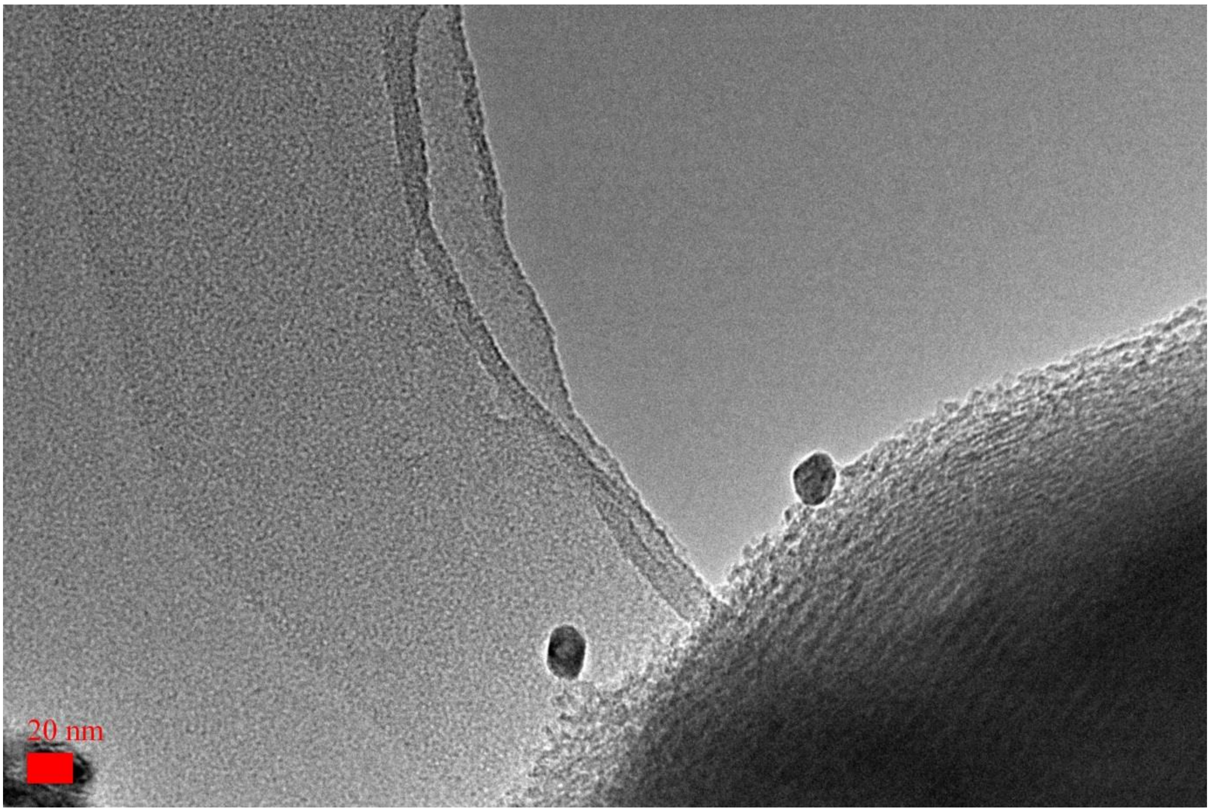

Figure S20. HRTEM image of FeKIT-5-5. Scale bar 20 nm.
